# Metabolic Control by DNA Tumor Virus-Encoded Proteins

**DOI:** 10.3390/pathogens10050560

**Published:** 2021-05-06

**Authors:** Martin A. Prusinkiewicz, Joe S. Mymryk

**Affiliations:** 1 Department of Microbiology and Immunology, Western University, London, ON N6A 3K7, Canada; mprusink@uwo.ca; 2 Department of Otolaryngology, Head & Neck Surgery, Western University, London, ON N6A 3K7, Canada; 3 Department of Oncology, Western University, London, ON N6A 3K7, Canada; 4 London Regional Cancer Program, Lawson Health Research Institute, London, ON N6C 2R5, Canada

**Keywords:** glycolysis, cellular respiration, mitochondria, oncovirus, metabolism, MYC, pRb, p53, oncoprotein, cancer

## Abstract

Viruses co-opt a multitude of host cell metabolic processes in order to meet the energy and substrate requirements for successful viral replication. However, due to their limited coding capacity, viruses must enact most, if not all, of these metabolic changes by influencing the function of available host cell regulatory proteins. Typically, certain viral proteins, some of which can function as viral oncoproteins, interact with these cellular regulatory proteins directly in order to effect changes in downstream metabolic pathways. This review highlights recent research into how four different DNA tumor viruses, namely human adenovirus, human papillomavirus, Epstein–Barr virus and Kaposi’s associated-sarcoma herpesvirus, can influence host cell metabolism through their interactions with either MYC, p53 or the pRb/E2F complex. Interestingly, some of these host cell regulators can be activated or inhibited by the same virus, depending on which viral oncoprotein is interacting with the regulatory protein. This review highlights how MYC, p53 and pRb/E2F regulate host cell metabolism, followed by an outline of how each of these DNA tumor viruses control their activities. Understanding how DNA tumor viruses regulate metabolism through viral oncoproteins could assist in the discovery or repurposing of metabolic inhibitors for antiviral therapy or treatment of virus-dependent cancers.

## 1. Introduction

Virally encoded oncogenes execute essential functions that allow DNA tumor viruses to reprogram cellular metabolism. Despite the variety of sizes and functionalities between the different oncoproteins encoded by different DNA tumor viruses, there are a few common targets that could be attractive candidates for future work that explores methods of targeting virally induced metabolic reprogramming for therapy. These include MYC, p53 and pRb/E2F, each of which has a unique set of metabolic targets. This review will outline how the host cell metabolic regulators MYC, p53 and pRb/E2F can, in turn, be targeted by DNA tumor virus proteins expressed by human adenovirus (HAdV), human papillomavirus (HPV), Epstein–Barr virus (EBV) and Kaposi’s sarcoma-associated herpesvirus (KSHV) during infection or in virus-dependent cancers.

### 1.1. MYC Metabolic Targets

MYC is a transcriptional regulator known to activate the transcription of genes involved in glycolysis, glutaminolysis, nucleotide synthesis and fatty acid synthesis [1,2,3,4]. As MYC is a transcription factor, this typically occurs through MYC-induced transcriptional upregulation of metabolic genes. This includes *LDHA*, which encodes an enzyme that converts pyruvate to lactate [2]. Another MYC-upregulated metabolic gene is *GLUT1*, which encodes a major glucose transporter [5]. The glutamine transporter encoded by *SLC1A5* is also upregulated by MYC [6]. As a result of increased MYC-induced glycolysis, the pentose phosphate pathway (PPP) is upregulated [7], and MYC is directly responsible for inducing transcription of the PPP enzyme-encoding gene *PRPS2* [8]. Many other nucleotide synthesis genes are upregulated by MYC [4]. In addition, a multitude of genes involved in fatty acid synthesis are upregulated by MYC, including *ACLY*, *ACACA*, *FASN* and *SCD* [1,9]. Finally, the processes of mitochondrial fusion and fission are reported to be under some level of control by MYC, which ultimately controls oxidative phosphorylation [10]. 

MYC itself can be regulated by a variety of pathways that can be aberrantly regulated in disease [11]. The PI3K/AKT signaling pathway increases MYC activity by inhibiting negative regulators of MYC [12]. The MAPK/ERK pathway also inhibits a negative regulator of MYC activity, MAD1 [13]. In terms of transcriptional regulation, TGF-β signaling through SMAD2/3 represses *MYC* gene transcription [14]. JAK/STAT signaling can regulate *MYC* transcription via a super enhancer region of the gene [15]. Likewise, estrogen receptors can bind *MYC* enhancers to regulate their transcription [16]. The Wnt signaling pathway can similarly regulate *MYC* transcription through Wnt response elements [17]. Finally, the Notch signaling pathway interacts with a complex that binds *MYC* enhancers [18].

Given that MYC impinges on so many metabolic pathways, understanding which DNA tumor viruses are capable of altering MYC activity could be important for understanding the mechanisms behind DNA tumor virus metabolic reprogramming.

### 1.2. p53 Metabolic Targets

Another protein commonly affected by viral oncoproteins is the tumor suppressor p53 encoded by the *TP53* gene. p53 is activated by cellular stress, including DNA damage, hypoxic conditions or oncoprotein activity [19,20,21,22]. These stresses trigger p53-mediated DNA damage repair, apoptosis, cell cycle arrest and metabolic pathways to counteract or mitigate the damage they cause [23,24,25,26,27]. p53 itself is regulated by the E3 ubiquitin ligase MDM2 to ensure that levels of the protein remain low under permissive, unstressed conditions [28]. In contrast, the phosphorylation of p53 at critical serine and threonine amino acid residues serves to stabilize the protein [29]. Due to its major role in counteracting cellular stress, p53 is one of the most frequently mutated genes in cancer [30]. 

Many viral oncoproteins inhibit the activity of p53, which, in turn, can increase the glycolytic activity of the infected cell [26]. This is because activated wild-type p53 can increase the activity of SCO2, which is involved in the assembly and regulation of the COX proteins that make up the mitochondrial cellular respiration complex IV [26]. Therefore, by inhibiting p53, viral oncoproteins inhibit mitochondrial function, which can subsequently trigger the upregulation of glycolytic pathways. p53 expression can also decrease the levels of the glucose transporters Glut1 and Glut4 [31], as well as the glucose metabolism enzyme PGM [32]. p53 increases expression of the glycolytic inhibitor TIGAR [33]. Understanding whether the inhibition of p53 by DNA tumor virus oncoproteins has any substantial effect on the resulting metabolic phenotype of the infected cell could further emphasize the importance of p53 in viral infection and virus-positive cancers.

### 1.3. pRb/E2F Metabolic Targets

DNA tumor viruses commonly target the activity of pRb, which is responsible for inhibiting the E2F family of transcription factors [34]. Like p53, pRb is typically regulated by E3 ubiquitin ligases, including MDM2, which cause the degradation of pRb [35]. MDM2 activity is triggered by the phosphorylation of pRb, regulated by proteins such as CDK4, CDKN2A and CCND1 [36,37]. Acetylation of pRb, induced by p300, inhibits the phosphorylation of pRb [38]. pRb can be SUMOylated, which leads to the expression of genes involved in cellular senescence [39]. Deubiquitinases, such as HAUSP, prevent pRb degradation [40]. 

In addition to regulating cell growth and proliferation through the cell cycle, E2F regulates the expression of a multitude of metabolic enzymes that are involved in glycolysis, the tricarboxylic acid (TCA) cycle, cellular respiration and nucleotide synthesis, helping to provide the energy and substrates necessary for the cell division cycle [41]. For example, E2F is responsible for regulating the expression of the glycolytic enzyme PFKFB [42,43]. E2F can also increase the expression of PDK4, which inhibits the entry of pyruvate into the TCA cycle, further promoting glycolysis [44]. Another way in which E2F expression leads to a metabolic phenotype that is more akin to cancer cells is by activating glutaminolysis, the process of metabolizing glutamine into TCA cycle metabolites [45,46]. E2F1 also plays a prominent role in inducing the expression of nucleotide synthesis genes [47]. As certain oncoproteins are capable of binding pRb, thus activating E2F, the ability of E2F to influence cellular metabolism is an important area of cancer research. 

## 2. Regulation of MYC by DNA Tumor Virus Oncoproteins

### 2.1. Regulation of MYC by HAdV

**Figure 1:**
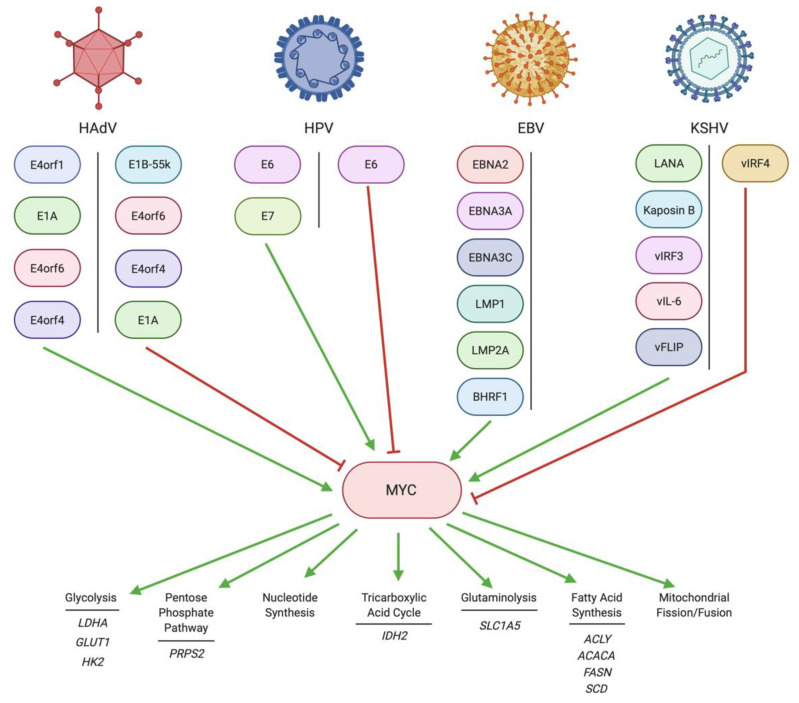
Regulation of MYC by DNA tumor virus oncoproteins. A number of HAdV proteins are reported to both positively and negatively regulate MYC, depending on the cellular and infection context. The HPV oncoprotein E6 is also reported to both activate and inhibit MYC activity, while E7 is a positive regulator of MYC. Multiple EBV viral proteins upregulate MYC activity. KSHV viral proteins generally activate MYC, although one KSHV protein, vIRF4, can downregulate MYC. MYC is a key regulator, primarily as a transcription factor, for many metabolic genes and pathways. Created with BioRender.com.

The most widely recognized oncoproteins in HAdV that can regulate MYC are E1A [48] and E1B [49] (Figure 1). E4orf1 [50] and E4orf6 [51] are two other HAdV proteins that have a role in MYC regulation (Figure 1). Recent advances in proteomics have identified that HAdV consistently upregulates MYC throughout infection [52]. 

In a series of elegant experiments, E4orf1 was identified to be a significant HAdV protein responsible for upregulating MYC expression during infection and contributing to the associated increases in glycolysis and glutaminolysis observed in HAdV-infected cells [53,54]. E4orf1 can upregulate MYC expression through a variety of signaling pathways that impinge on MYC activity, namely those including either EGFR, INSR/IGF1R or PI3K [55]. 

The HAdV oncoprotein E1A is reported to influence MYC activity through the TRRAP protein [56]. E1A can also stabilize MYC itself [57]. Additionally, a complex between E1A and p400 was found to target MYC and bring both p400 and MYC to the promoter regions of genes and increase their transcription [58]. E1A has also been reported to disrupt p300-mediated repression of MYC [59]. All of these interactions between E1A and MYC could account for our recent findings, which illustrate that glycolysis can be upregulated by E1A [60]. While there are examples of select regions of E1A being able to inhibit MYC through p300/CBP or TRRAP [61], this could suggest that, rather than completely abolishing MYC activity, E1A may selectively modulate which MYC targets are transcribed depending upon which regions of E1A are available for interaction with MYC and through which complexes the E1A/MYC interaction occurs. 

The HAdV protein E4orf6 can influence the upregulation of MYC in two ways. One is via stabilization of the *MYC* transcript, presumably leading to greater production of MYC [62]. A second mechanism by which E4orf6 can influence MYC activity is through further stabilization of the interaction between E1A and MYC [53]. Additionally, both E4orf6 and E1B-55K have been reported to downregulate MYC expression [57], a contradictory function for E4orf6. Speculatively, different outcomes of E4orf6 effects on MYC could occur at different stages of infection. This could depend on the HAdV type under investigation or may be dependent on which other viral or host cell proteins are available for interacting with E4orf6 in the context of different experimental systems.

In contrast to most of the HAdV products described above, E4orf4 inhibits MYC during infection [63]. It should be noted that E4orf4 is not defined as a HAdV oncoprotein, and this study used HEK293 cells, which already express E1A and E1B [64], thus potentially influencing the results. In a contradiction to the report above, E4orf4 has also been found to cause an increase in MYC protein levels [65]. However, this study examined the E4orf4 protein in isolation as a vector without the full complement of HAdV proteins that would be expressed during infection, which could explain this contrasting observation. 

Taken together, the control of MYC expression, localization and activity is clearly an important task during HAdV infection, given that so many viral proteins target this cellular hub to reprogram gene expression in the host cell. It is clear that at least some of these impact cellular metabolism [60,66], but much remains to be learned.

### 2.2. Regulation of MYC by HPV

Numerous reports have identified functional effects of the HPV E6 and E7 oncoproteins on MYC (Figure 1). Both E6 and, albeit less efficiently, E7 have been reported to upregulate MYC [67,68]. With respect to E6, the association with MYC has been investigated primarily in the context of upregulation of hTERT expression. Several studies found that MYC physically associated with E6 and was critical for E6 activation of hTERT transcription [68,69]. However, it has become relatively clear that E6 does not induce hTERT transcription simply by inducing expression of MYC, but rather the involvement of other host cell proteins is required [70]. Somewhat confusingly, it was reported that E6 can also stimulate the degradation of MYC [71]. These differences may be attributed to cell type, as this contradictory result was observed in a neuroblastoma cell line [71]. This does not represent the tissue type typically infected by HPV, which is better represented by human foreskin keratinocytes [68]. Perhaps the most convincing evidence that E6 stimulates MYC activity, and that this overexpression is crucial to E6-mediated transformation by HPV, is the ability of MYC overexpression to immortalize cells in cooperation with E7. Thus, E7 and MYC cooperate in a similar manner, but not identical to E6 and E7, to induce immortalization [72]. 

As for the E7 oncoprotein, it is possible that HPV18 E7 can interact with MYC to promote MYC-regulated transcription [73,74]. It is also possible that E7 can promote MYC activity indirectly, as a study utilizing HPV16 E7 found that E7 could inhibit MIZ1, a negative regulator of MYC function [75]. The result of this was increased MYC function. Perhaps somewhat contradictorily, two studies of HPV+ head and neck squamous cell carcinoma tissue samples did not identify an association between the cancer and increased MYC expression [76,77]. However, another study of patient tissues found that HPV integration within the tonsillar crypt was associated with MYC amplification and overexpression [78]. The upregulation of MYC by HPV, predominantly enacted through the E6 oncoprotein [68], could serve to increase the expression of metabolic genes involved in glycolysis, glutaminolysis and nucleotide synthesis [79], but this remains to be conclusively determined.

### 2.3. Regulation of MYC by EBV

In B cell lymphomas, EBV drives MYC expression in a variety of ways (Figure 1). Perhaps the most common EBV-induced change to MYC expression is in the form of a common *MYC* gene translocation event in EBV-positive Burkitt’s lymphoma [80]. In this translocation event, *MYC* is translocated to the immunoglobulin heavy locus gene, *IGH*, where it comes under the control of the highly active *IGH* regulatory region [81]. This leads to constitutive expression of MYC in Burkitt’s lymphoma [81,82]. However, the expression of multiple EBV viral proteins has also been associated with MYC expression in B cell cancers. EBNA2 was noted to activate *MYC* transcription in primary B lymphocytes [83]. EBNA2 can also upregulate MYC protein levels [84]. There is evidence that EBNA2 can increase interactions between enhancers and promoters upstream of *MYC* through the chromatin regulator SMARCA4 [85]. EBNA2 can bind DNA regions termed EBV-super enhancers, in which a complex of EBV latent proteins, including EBNA3A and EBNA3C, as discussed below, can increase the expression of host cell genes, such as *MYC* [86]. EBNA2 may also increase the expression of non-coding enhancer RNAs (eRNAs) that regulate *MYC* transcription [87]. EBNA2-driven MYC expression can upregulate mitochondrial one-carbon metabolism, specifically the de novo synthesis of serine, which contributes to nucleotide synthesis, mitochondrial NADPH production and production of the antioxidant glutathione [88]. Other metabolic pathways upregulated by EBNA2-driven expression of MYC in B cells include cholesterol and lipid biosynthesis [89,90]. 

Two other EBNA proteins, EBNA3A and EBNA3C, also contribute to MYC expression. Both EBNA3A and EBNA3C contribute to the EBV super enhancer structure that ensures the presence of transcription factors at the *MYC* transcription start site via enhancer–promoter looping [91]. Additionally, EBNA3C stabilizes the interaction between MYC and MYC target promoters, essentially increasing MYC activity [92]. To further augment MYC expression, EBNA3A and EBNA3C cooperate to epigenetically repress *BCL2L11* expression, which encodes a negative regulator of MYC. *BCL2L11* expression can be induced by MYC in the absence of EBNA3A and EBNA3C in a negative feedback mechanism [93,94,95]. 

The EBV latent membrane proteins also play a role in increasing MYC expression in EBV-driven cancers. In B cells, LMP1 can increase *MYC* transcription and resulting MYC protein expression via the JAK/STAT pathway [96]. STAT3 in particular is upregulated in EBV-positive nasopharyngeal carcinoma cell lines [97,98]. Additionally, in EBV-induced nasopharyngeal carcinoma, LMP1 facilitates increased interactions between MYC and the MYC target genes *HK2* and *IDH2*, which encode enzymes in glycolysis and the TCA cycle, respectively [99,100,101]. This MYC stabilization occurs through an LMP1 signaling pathway that involves PI3K/AKT/GSK3β and FBXW7 signaling [100,101]. The importance of the LMP1-mediated MYC activation pathway was emphasized by a study in which the histone deacetylase inhibitor romidepsin was found to be cytotoxic to an EBV-positive diffuse large B-cell lymphoma in both a cell culture and a mouse xenograft model by reducing the expression of both LMP1 and MYC [102]. 

In addition to causing increased MYC expression and activity, LMP1 downregulates the α-isoform of the MYC repressor PRDM1, which further accentuates LMP1-driven MYC activation [103]. In a similar manner, LMP2A can facilitate MYC activity by enhancing the degradation of the MYC inhibitor and tumor suppressor, CDKN1B [104]. LMP2A can also increase the translation of MYC, but not the transcription of *MYC*, through the PI3K/AKT/mTOR signaling pathway [105]. Finally, the EBV pro-survival BCL-2 homologue, BHRF1, enhanced MYC activity in a mouse model of Burkitt’s lymphoma [106]. EBV appears to tightly regulate MYC in infected B cells to modulate lytic reactivation that would otherwise occur in the absence of MYC [107].

Interestingly, the regulation of MYC by EBV oncoproteins appears to be relatively minimal in EBV-associated gastric cancer (EBVaGC). At most, MYC may be upregulated in EBVaGC during its early stages [108]. However, at later times during the progression of EBVaGC, MYC is either downregulated [108,109] or MYC expression does not correlate with EBV expression at all [110,111,112]. These findings suggest that MYC may not have a role in EBV-induced metabolic alterations associated with EBVaGC. However, in most cases, especially in B cell-related cancers, MYC upregulation by EBV drives serine catabolism for nucleotide synthesis, glycine metabolism for glutathione involved in redox metabolism, and lipid synthesis [113].

### 2.4. Regulation of MYC by KSHV

KSHV is capable of regulating MYC function through a number of its latent proteins [114] (Figure 1). The KSHV protein LANA upregulates MYC function through inactivation of GSK-3 [115,116,117]. The inactivation of GSK-3 is achieved in two ways. Firstly, GSK-3 is removed from the cytoplasmic β-catenin destruction complex [118], which allows β-catenin to activate transcription of *MYC* [117]. Secondly, inactivation of GSK-3 by LANA leads to decreased GSK-3-mediated phosphorylation of Thr58 on MYC, thus stabilizing MYC by preventing its degradation through ubiquitination [116,119]. Additionally, ERK1 is stabilized by LANA, which stimulates the phosphorylation of MYC at Ser62, increasing the transcriptional activity of MYC on its gene targets [116]. 

MYC can form a complex with kaposin B, which is another KSHV latent protein, to modulate the expression of a wide variety of host microRNAs [120], some of which, such as miR-210 [121,122,123,124,125], miR-3188 [126], miR-483-3p [127], miR-3197 [128], miR-423-5p [129], let-7f-5p [130], miR-372-3p [131], miR-9-5p [132,133], miR-489-3p [134], miR-1271-5p [135], miR-7-5p [136] miR-942-3p [137] and miR-153-3p [138,139], have been linked to metabolism in other diseases. In addition, the KSHV latent protein vIRF3 can stimulate the transcriptional activity of MYC by interacting with Skp2, a transcriptional cofactor that stabilizes and upregulates MYC activity [140]. Another way in which vIRF3 stimulates MYC activity is by inhibiting a negative regulator of MYC, PFDN5 [141]. Finally, vIRF3 may interact with the promoter region of MYC targets, as was demonstrated for *CDK4*, to increase histone H3 acetylation, thereby encouraging MYC-mediated transcription of the target [141]. 

The KSHV latent protein vIRF4 has an inhibitory effect on MYC [142], which assists the latently infected cell to enter the lytic phase of KSHV replication [143]. This MYC-regulated switch between the latent and lytic phases of infection is a parallel between KSHV and EBV infections [143]. vIRF4 appears to suppress MYC activity by limiting the amount of eRNAs produced by cellular IRF4, thereby limiting the super enhancer function of IRF4 and lowering MYC expression [144]. The control of eRNA expression to modulate *MYC* transcription is another parallel between KSHV and EBV. 

The KSHV protein vIL-6, while not directly responsible for upregulating *MYC* transcription or MYC protein expression, could enhance the oncogenic activities of MYC in a KSHV-associated cancer mouse model [145]. Similarly, the latent protein vFLIP appeared to accelerate lymphoma development in conjunction with MYC in a mouse model [146]. 

KSHV can also influence glutaminolysis through MYC as MYC/MAX and MLXIP/MLX heterodimers that induce expression of the glutamine transporter SLC1A5 [147]. One of the main results of KSHV-mediated upregulation of MYC function is the upregulation of glutamine metabolism, upon which KSHV is particularly reliant [147]. 

## 3. Regulation of p53 by DNA Tumor Virus Oncoproteins

### 3.1. Regulation of p53 by HAdV

**Figure 2:**
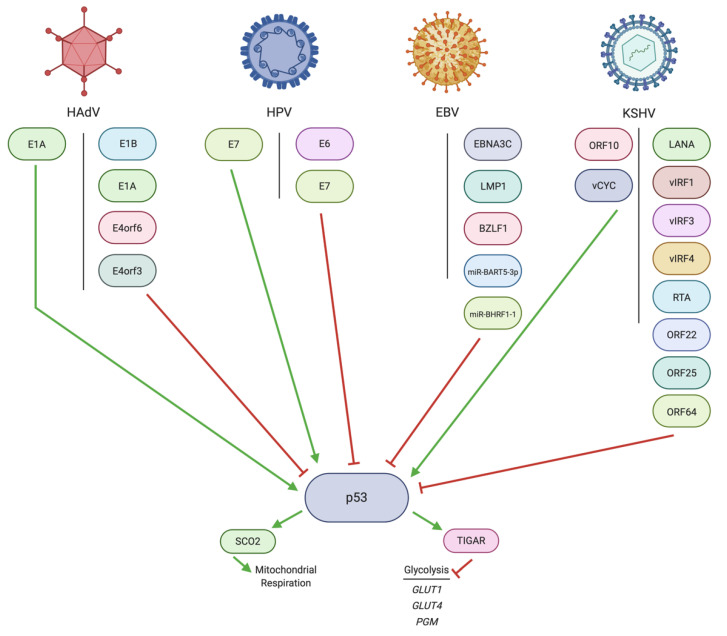
Regulation of p53 by DNA tumor virus oncoproteins. Most HAdV proteins inhibit p53. E1A generally induces p53 activity, but it can also inhibit p53. Like E1A, the HPV protein E7 induces p53 activity, but it has also been reported to inhibit p53. E6 is a p53 inhibitor. Three EBV proteins and two EBV miRNAs can inhibit p53. While most KSHV proteins limit p53 activity, two KSHV proteins, ORF10 and vCYC, can upregulate p53 function. p53 is a pro-apoptotic protein and tumor suppressor that can inhibit glycolysis and encourage mitochondrial respiration. Created with BioRender.com.

The E1B oncoproteins are the HAdV proteins most directly responsible for binding to and inhibiting the transcriptional activation activity of p53 [148,149,150] (Figure 2). The E1B-55K isoform of E1B can bind p53 in a complex that involves E4orf6 [151,152]. It appears that this complex can sequester p53 away from the nucleus [153,154]. In addition, E1B-55K can SUMOylate p53 to influence its function [155]. p53 SUMOylation by E1B-55K leads to tethering and colocalization of p53 to E1B-55K, thus decreasing the nuclear mobility of p53 and maximally inhibiting its function [156]. 

E1B-19K can directly bind p53 within the mitochondria and inhibit mitochondrially mediated p53 induction of apoptosis [157]. E1B-19K can also bind BAK within the mitochondria to competitively inhibit binding by p53, thus blocking apoptosis [157]. 

The ability of E1A to interact with and affect p53 is more nuanced. It appears that E1A induces apoptosis through p53 and even triggers p53 accumulation [158,159,160]. The accumulation of p53 triggered by E1A appears to play a role in late viral protein production [161]. For example, p53 contributes to activation of the adenovirus L4 promoter within the major late transcription unit [162]. However, the beneficial contributions of p53 to HAdV replication are somewhat controversial [163]. 

E1A has also been reported to inhibit the transcriptional activation of target genes by p53 and repress its activity [164,165]. It is possible that this repression occurs through the formation of a complex between E1A, FUBP1 and p53, which serves to sequester p53 away from gene promoter regions [166]. Additionally, E1A and either mutant p53 or an absence of p53 entirely have been reported to transform cells [167,168], which would presumably lead to the induction of cancer-related metabolic pathways. 

It is possible that these contradictory results regarding whether E1A stabilizes and activates p53 or represses its activity could be attributed to a variety of factors. For example, E1A may induce p53 in HAdV5-transformed cells or when the E1A protein is expressed alone as a vector within a cell [159]. The induction of p53 may be dependent on the E1A isoform, with the 12S isoform inducing apoptosis via p53, while the 13S isoform does not [158]. Finally, it could be that E1A inhibits p53 activity in HAdV infection of human cells, where the full extent of viral gene expression and processes can occur [166].

Two of the HAdV E4 proteins have been reported to contribute to p53 inhibition. E4orf6 inhibits the transcriptional activity of p53 [169]. The reduction in p53 activity enacted by E4orf6 is at least partially due to a complex formed by E4orf6 and Cul5-Elongin B-Elongin C E3 ubiquitin ligase that targets p53 for degradation [170]. E4orf6 can induce neddylation of Cul5, which encourages the nuclear localization of Cul5, where it can then interact with p53 [171]. Neddylation is the addition of a ubiquitin-like protein NEDD8 to target proteins, which can enact a multitude of functions from protein degradation to relocalization [172]. This complex appears to function optimally when the E4orf6 ligase complex includes E1B-55K [173]. In this complex, E1B-55K increases the amount of p53 that can interact with E4orf6 and the ligase [173]. E4orf3 can induce inhibitory H3K9 methylation of the promoters to which p53 would bind [174]. 

The ability of p53 to influence metabolism is well established [175]. The most notable changes enacted by p53 include downregulation of glycolysis and lipid biosynthesis and upregulation of mitochondrial respiration, beta-oxidation and the PPP [175]. However, whether p53 has a role in the metabolic changes that occur in context of HAdV infection remains to be conclusively identified.

### 3.2. Regulation of p53 by HPV

The HPV E6 oncoproteins, especially those of the most oncogenic types, HPV16 and HPV18, are responsible for modulating p53 activity by binding to and inducing the degradation of p53 [176,177,178] (Figure 2). The mechanism by which this occurs is thought to involve a complex between E6 and a host cell ubiquitin ligase, E6AP. [179]. When this complex binds p53, it is capable of inducing p53 degradation [179]. Interestingly, ubiquitination of E6 is required for this complex to degrade p53 successfully [179]. The efficiency of this complex appears to vary with HPV type [180]. Another mechanism utilized by E6 to contribute to the degradation of p53 involves nuclear export of a protein involved in heterochromatin formation, HP1γ [181]. HP1γ inhibits expression of UBE2L3, which assists E6-mediated degradation of p53 [181]. 

In contrast, the HPV E7 oncoprotein was reported to stabilize p53, which promoted apoptosis of infected cells [182]. E6 can contribute to the bypassing of this apoptotic induction by E7, which bears some resemblance to the opposing activities of HAdV E1A and E1B. However, it has been reported that E7 may inhibit p53 transcriptional activity [183]. One mechanism through which this occurs is by E7 inhibition of the p53-mediated DREAM pathway [184]. This implies that E7 could influence the changes in cellular metabolism that occur as a result of p53 inhibition. Neither of these results were generated in the context of HPV infection; however, differing cell lines and genetic vectors were used to deliver E7 into the cells. The study that found that E7 stabilizes p53 used a retroviral vector in primary IMR-90 fetal lung cells [182], while the studies that observed an inhibition of p53 activity by E7 used a plasmid vector and either osteosarcoma [183] or colon carcinoma cell lines. It is possible that these differences may explain why divergent effects of E7 on p53 were observed [184].

It is quite possible that HPV can upregulate glycolysis through the downregulation of p53 by the mechanisms discussed above. It would be interesting to see whether these and other metabolic hallmarks of HPV-induced cancers [185] are definitively driven by the differential expression of p53 when compared to corresponding HPV-negative cancers and non-cancerous tissues.

### 3.3. Regulation of p53 by EBV

EBV can influence the function of p53 through a variety of viral oncoproteins (Figure 2). These include the EBV latency protein EBNA3C, which has been noted to inhibit p53 DNA binding activity [186,187]. EBNA3C is thought to both block the transcriptional activity of p53 directly and encourage the degradation of p53 through the MDM2 protein as a result of this interaction [188]. EBNA3C also inhibits p53 by inducing the formation of a complex with the host cell DDX20 protein [189]. This complex prevents p53 binding to DNA and, in turn, reduces expression of the pro-apoptotic genes regulated by p53 [189]. Additionally, EBNA3C can interact with proteins that regulate p53, such as the positive regulators ING4 and ING5 [188]. EBNA3C forms a complex with either ING4 or ING5 and p53 that inhibits p53 activation by these two ING proteins [188]. 

LMP1 is another EBV latency protein and oncoprotein that can inhibit the activity of p53 [190,191,192,193]. There are reports of LMP1 achieving this through p53 ubiquitination at K48 or K63, the result of which is uncontrolled proliferation and an inhibition of apoptosis [194]. Interestingly, p53 also induces LMP1 expression, which is an example of a feedback loop in which the virus may be directly responding to the activity of tumor suppressor pathways [195]. 

Finally, the EBV immediate early lytic protein BZLF1 can inhibit p53 transactivation of its targets [196]. This is partially due to BZLF1-induced degradation of p53 through an ECS E3 ubiquitin ligase complex [197,198], a parallel with how E1B-55K and E4orf6 can also degrade p53 through an E3 ubiquitin ligase complex in HAdV infection. 

In addition to protein-mediated effects, the EBV-encoded miR-BHRF1-1 miRNA can downregulate p53 expression in nasopharyngeal cancer cell lines by binding to the 3′ UTR of *TP53* mRNA [199]. miR-BHRF1-1 is also associated with downregulation of *TP53* in peripheral blood mononuclear cells from EBV-positive chronic lymphoid leukemia patients [200]. Another EBV-encoded miRNA that can target and inhibit p53 is mIR-BART5-3p [201]. mIR-BART5-3p can inhibit p53 in two ways. One way is through targeting the 3′ UTR of *TP53* mRNA, which then leads to a measurable downregulation of expression of p53-regulated genes, including *CDKN1A*, *BAX* and *FAS* [201]. miR-BART5-3p can also contribute to the degradation of p53 protein [201]. 

It would be interesting to explore whether there is a direct link between the expression of each of these oncoproteins or viral miRNAs within EBV-positive cancer cell lines, the resulting expression of p53 and glycolytic upregulation or other associated metabolic changes.

### 3.4. Regulation of p53 by KSHV

The KSHV latency protein LANA can interact with p53 to repress cell death pathways mediated by p53 [202] (Figure 2). Additionally, LANA is a component of the EC_5_S E3 ubiquitin ligase complex that targets p53 for degradation by polyubiquitination, thus limiting the activity of p53 by another mechanism [203]. The interaction of LANA with EC_5_S could be mediated through its interactions with the complex components CUL5 and RBX1 [204]. Another host cell kinase, AURKA, is noted to increase the anti-p53 activities of LANA [205]. The inhibition of p53 by LANA can also cause increased chromosomal instability [206], perhaps contributing to the formation of KSHV-positive cancers. It is possible that the interaction of LANA with p53 is based on the stoichiometric availability of these two proteins within the infected cell [207]. It has also been suggested that the inhibitory interaction between LANA and p53 is enhanced by KSHV-encoded miRNAs [208].

The KSHV vIRF proteins can inhibit p53 function in KSHV-infected cells and models of B cell lymphomas. vIRF1 can associate with p53 and repress its transcriptional activity [209,210]. vIRF3 interaction with p53 also inhibits p53-mediated apoptotic pathways [211]. This interaction involves vIRF3 binding to the DNA binding domain of p53, which inhibits the phosphorylation of serines 15 and 20, which are key residues for the apoptotic functions of p53 [212]. vIRF3 also increases p53 ubiquitination, targeting it for proteasomal degradation [213]. Furthermore, vIRF3 inhibits SUMOylation of p53 by SUMO2 [214]. SUMOylation of p53 by SUMO2 is thought to enhance its activity [39,215,216]. This is in contrast to the SUMOylation of p53 by SUMO1 during HAdV infection as noted above, which has an inhibitory effect on p53 activity due to the tethering of p53 to E1B-55K [156]. vIRF4 can also inhibit p53 by increasing the stability of MDM2, which leads to an increase in p53 ubiquitination and degradation, thus inhibiting p53 pro-apoptotic activity [217].

Proteins involved in the lytic stage of KSHV infection can also inhibit p53 function. For example, the KSHV RTA protein, responsible for the switch between the latent and lytic cycles of KSHV replication [218], can repress both the transcriptional and apoptotic activities of p53 through an interaction with the CBP transcriptional coactivator [219]. Three other structural proteins expressed in the late lytic phase appear to inhibit p53 [220]. These are envelope glycoprotein H (ORF22), major capsid protein (ORF25) and large tegument protein deneddylase (ORF64) [220]. It is possible that these proteins serve to inhibit p53 during the entrance phase of infection in a newly infected cell rather than inhibiting p53 during the end of lytic KSHV replication [220].

There are two KSHV proteins that can augment p53 expression and stability. One protein, known as ORF10, increases the prevalence of the phosphorylation of p53 at serine 15, associated with increased p53 expression, and can reduce p53 ubiquitination [221]. The second KSHV protein that can stabilize p53 is vCYC, which stabilizes p53 through CDK9-mediated phosphorylation of p53 at serine 33 [222]. It is likely that the stabilization of p53 by these KSHV proteins mediates the transition to the lytic phase of the KSHV replication cycle [223].

While KSHV is reported to alter the activity of many metabolic pathways [224], including those that are regulated by p53 [225], whether the interaction between KSHV and p53 is the direct cause of any of these metabolic changes remains to be explored.

## 4. Regulation of pRb/E2F by DNA Tumor Virus Oncoproteins

### 4.1. Regulation of pRb/E2F by HAdV

**Figure 3:**
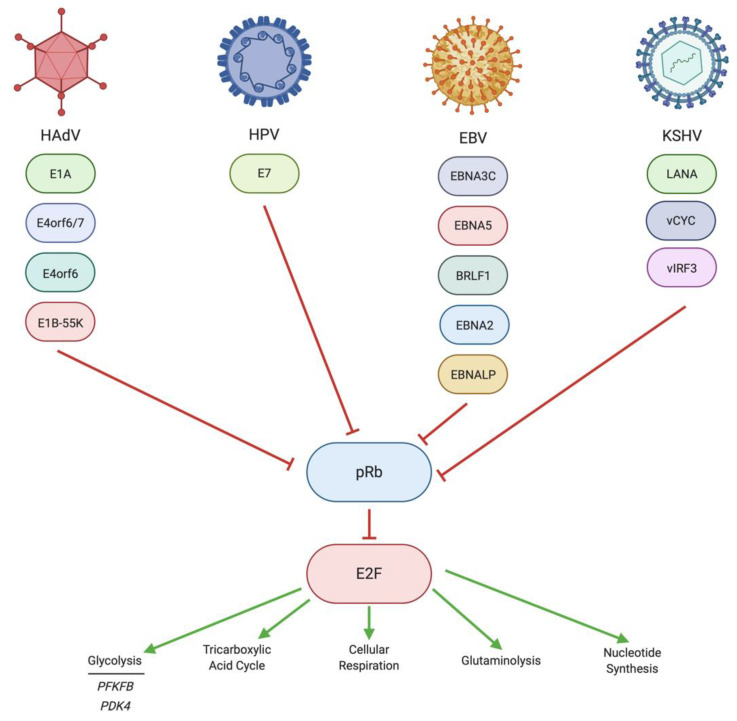
Regulation of pRb/E2F by DNA tumor virus oncoproteins. Each of the DNA tumor viruses discussed in this review encodes proteins that can inhibit pRb suppression of E2F function. This allows for the upregulation of a wide variety of E2F targets. E2F, when activated, can alter the function of a wide variety of metabolic pathways. Created with BioRender.com.

The HAdV oncoprotein that has a predominant interaction with pRb is E1A [226] (Figure 3). E1A can bind pRb directly to release E2F inhibition [227], thus greatly increasing the transcription of a variety of genes, including metabolism-related genes. This high affinity interaction is mediated by an LXCXE motif as well as a second site that mimics the portion of the E2F activation domain that normally interacts with pRb [228]. However, if E1A is bound to both pRb and p300, this can have an inhibitory effect on the transcription of other metabolism-related genes that are not transcribed by E2F due to the ability of this complex to lead to chromatin hypoacetylation and condensation [229]. Mechanistically, in this case, p300 linked to E1A may acetylate pRb in the complex, preventing inhibitory pRb phosphorylation [229]. Another mechanism by which E1A could inhibit pRb is by binding the LXCXE motif on pRb, which may sterically block access to a SUMOylation site on pRb [230]. SUMOylation of pRb is associated with relief of E2F repression [230]. The role of SUMOylation of pRb in the context of adenovirus infection is still not completely understood [231], and therefore, while SUMOylation of pRb may be important in uninfected cells, it is possible that HAdV can bypass this modification entirely. 

Another HAdV protein, E4orf6/7, can displace pRb on E2F1, increasing transcriptional activation by E2F1 [232]. In addition to displacing pRb, E4orf6/7 was found to be involved in the nuclear localization of another E2F family member, E2F4 [233]. Likewise, E1A has been shown to activate E2F family members independently of pRb displacement. The 13S isoform of E1A can interact with E2F4 through a complex containing DP1, which leads to increased transcriptional activity of E2F4 [234]. Interestingly, this function is limited to the 13S isoform of E1A, and our study of the influence of E1A isoforms on metabolism showed that the 13S isoform of E1A was responsible for regulating distinct aspects of cellular metabolism [60]. 

In another potentially redundant mechanism, the E3 ubiquitin ligase complex formed from E4orf6/E1B55K can also displace pRb from E2F family members, thereby permitting these transcription factors to transcribe their target genes [235]. The E4orf6/E1B-55k ligase complex can also enhance the E2F-releasing activities of E1A [236].

Studies examining the role of E1A-mediated release of E2F suppression by pRb on metabolism are limited. An analysis of published RNA-Seq datasets utilizing mutant E1A deficient for pRb binding [229] indicated that this interaction is indeed responsible for altering the expression of metabolism-related genes, particularly in nucleotide metabolism and glycerolipid metabolism, as reviewed by ourselves in [66]. 

### 4.2. Regulation of pRb/E2F by HPV

The E7 oncoprotein of HPV is primarily responsible for influencing pRb (Figure 3), especially at early stages of infection [237,238,239]. E7 induces the degradation of pRb [240], but it is also capable of simply displacing pRb from E2F family members to induce DNA synthesis pathways [241]. E7 from high-risk HPV is especially effective at degrading pRb protein, without affecting *RB1* mRNA expression [242]. However, like E1A, E7 inhibits SUMOylation of pRb [230]. As with E1A, SUMOylation of pRb is probably not required for the repression of pRb inhibitory functions by E7. Influencing pRb activity does not appear to be a major role for any of the other HPV-encoded proteins, including the E6 HPV oncoprotein. As with p53, the role of pRb/E2F in regulating metabolism in the context of HPV infection or HPV-positive cancers has not been thoroughly elucidated. Any effects of pRb/E2F on metabolism during HPV infection or in HPV-positive cancers, such as a theoretical upregulation of glutaminolysis or nucleotide metabolism, have been deduced based on studies performed on pRb/E2F in isolation and remain to be conclusively demonstrated in the context of HPV infection and HPV-positive cancers.

### 4.3. Regulation of pRb/E2F by EBV

A variety of EBV-encoded viral proteins are capable of inducing E2F expression or activity. One example is the immediate-early EBV protein BRLF1, which induces expression of E2F itself [243,244]. Some of the EBV-encoded EBNA proteins are also capable of binding pRb, thus freeing E2F activity. Indeed, both EBNA3C [245] and EBNA5 [246] can bind pRb to induce E2F-dependent gene expression (Figure 3). Additionally, EBNA3C can bind E2F1 directly at the N-terminal region of E2F1 to block the pro-apoptotic activities of E2F1, which still allows the proliferation-promoting activities of E2F1 to occur [247]. Another mechanism by which EBNA3C regulates the transcription activities of E2F1 is through a complex formed between EBNA3C, E2F1 and another E2F family member, E2F6 [248]. This serves to further repress E2F1-mediated expression of genes that inhibit abnormal proliferation [248]. It also appears that interactions between either EBNA2, EBNA3C or EBNALP and E2F/pRb/HDAC complexes may also be involved in regulating the expression of anti-proliferative genes [249]. 

The pRb/E2F complex is responsible for regulating a wide range of metabolic processes, including glycolysis, central carbon metabolism, redox metabolism and mitochondrial biogenesis [41,250]. However, how EBV interacts with pRb/E2F to directly influence these metabolic pathways still remains generally speculative based upon the functions of pRb/E2F alone [251].

### 4.4. Regulation of pRb/E2F by KSHV

Unlike its multifaceted interactions with MYC and p53, only a few KSHV-encoded latent proteins appear to interact with pRb (Figure 3). LANA targets pRb, freeing E2F to activate transcription [252]. vCYC can promote the phosphorylation of pRb, leading to its inactivation and, again, allowing E2F-mediated transcription to occur [253]. In this case, phosphorylation of pRb is mediated by cellular CDK6, which is activated by vCYC [253]. Finally, vIRF3 can bind to pRb and contains an LXCXE motif similar to those found in HAdV E1A and HPV E7 [254]. However, this motif is not required for pRb binding by vIRF3 [254]. Instead, the LXCXE motif is required for vIRF3 inhibition of pRb SUMOylation, which blocks the tumor suppressor functions of pRb [254]. 

Like HPV and EBV, studies directly linking the effects of KSHV on the pRb/E2F complex and the resulting effects of that interaction on metabolism are limited. It is well established that KSHV can upregulate glycolysis, glutaminolysis and fatty acid synthesis while contributing to the downregulation of oxidative phosphorylation during KSHV infection [255]. Despite this, it has not been conclusively shown which of these pathways are indeed regulated by an interaction between KSHV and pRb/E2F [251].

## 5. Conclusions

This review outlined how four different DNA tumor viruses—HAdV, HPV, EBV and KSHV—all target three specific host cell regulatory proteins known to influence host cell metabolism. The importance of these proteins, namely MYC, p53 and pRb/E2F, in promoting cell growth and oncogenesis overshadows their ability to regulate host cell metabolism. Nevertheless, their important role in controlling cell metabolism should not be overlooked. For example, in MYC-driven cancers, targeting of certain glycolytic enzymes upregulated by MYC represented vulnerabilities that are not present in cells with non-dysregulated levels of MYC and are potentially targetable for treatment [256,257]. It is possible that cancers caused by DNA tumor viruses exhibit similar metabolic alterations, unwittingly creating novel vulnerabilities in the infected cells [258,259]. Targeting a viral oncoprotein, its cellular regulatory protein effector or the resulting upregulated metabolic pathway could be interesting avenues in which to explore synthetic lethality for the treatment of viral diseases or the cancers associated with DNA tumor virus infections [258].
